# Indirect comparisons of efficacy of zanubrutinib versus orelabrutinib in patients with relapsed or refractory chronic lymphocytic leukemia/small lymphocytic lymphoma or relapsed or refractory mantle cell lymphoma

**DOI:** 10.1007/s10637-023-01376-1

**Published:** 2023-07-08

**Authors:** Yuqin Song, Keshu Zhou, Shenmiao Yang, Jianda Hu, Dehui Zou, Sujun Gao, Ling Pan, Tingyu Wang, Haiyan Yang, Huilai Zhang, Daobin Zhou, Jie Ji, Wei Xu, Ru Feng, Jie Jin, Fangfang Lv, Haiwen Huang, Xiaosi Fan, Sheng Xu, Jun Zhu

**Affiliations:** 1 Department of Lymphoma, Peking University Cancer Hospital & Institute (Beijing Cancer Hospital), No. 52 Fucheng Road, Haidian District, Beijing, 100142 China; 2 Department of Hematology, Affiliated Cancer Hospital of Zhengzhou University, Henan Cancer Hospital, Zhengzhou, China; 3 Department of Hematology, Peking University People’s Hospital, Peking University Institute of Hematology, Beijing, China; 4 Department of Hematology, Fujian Institute of Hematology, Fujian Medical University Union Hospital, Fuzhou, China; 5 State Key Laboratory of Experimental Hematology, Institute of Hematology & Blood Diseases Hospital, Chinese Academy of Medical Sciences and Peking Union Medical College, Tianjin, China; 6 Department of Hematology of Cancer Center, The First Hospital of Jilin University, Changchun, China; 7 Department of Hematology, West China Hospital of Sichuan University, Chengdu, China; 8 Department of Lymphoma, The Cancer Hospitalof the, University of Chinese Academy of Sciences (Zhejiang Cancer Hospital), Hangzhou, China; 9 Department of Lymphoma, Tianjin Medical University Cancer Institute and Hospital, National Clinical Research Center for Cancer, Key Laboratory of Cancer Prevention and Therapy, Tianjin’s Clinical Research Center for Cancer, Tianjin, China; 10 Department of Hematology, Union Medical College Hospital, Chinese Academy of Medical Sciences & Peking Union Medical College, PekingBeijing, China; 11 Department of Hematology, The First Affiliated Hospital of Nanjing Medical University, Jiangsu Province Hospital, Nanjing, China; 12 Department of Hematology, Nanfang Hospital of Southern Medical University, Guangzhou, China; 13 Department of Hematology, The First Affiliated Hospital, Zhejiang University College of Medicine, Hangzhou, China; 14 Department of Medical Oncology, Fudan University Shanghai Cancer Center, Shanghai, China; 15 Department of Hematology, The First Affiliated Hospital of Soochow University, Suzhou, China; 16 BeiGene (Beijing) Co., Ltd, Beijing, China

**Keywords:** R/R CLL/SLL, R/R MCL, Zanubrutinib, Orelabrutinib, Indirect treatment comparison

## Abstract

We conducted two indirect comparisons to estimate the efficacy of zanubrutinib versus orelabrutinib in Chinese patients with relapsed or refractory (R/R) chronic lymphocytic leukemia/small lymphocytic lymphoma (CLL/SLL) or R/R mantle cell lymphoma (MCL). An unanchored matching-adjusted indirect comparison (MAIC) was performed in R/R CLL/SLL patients. Individual patient data from zanubrutinib trial (BGB-3111-205) were adjusted to match the aggregated data from the orelabrutinib trial (ICP-CL-00103). A naïve comparison was performed in R/R MCL for the different response assessment methodology and efficacy analysis set between the zanubrutinib (BGB-3111-206) and orelabrutinib (ICP-CL-00102) trials. Efficacy outcomes included ORR and PFS. In R/R CLL/SLL patients, after matching, IRC-assessed ORR was comparable (86.6% vs. 92.5%; risk difference, -5.9% [95% CI: -15.8%-3.8%]); IRC-assessed PFS was similar with a favorable trend in zanubrutinib over orelabrutinib (HR, 0.74 [95% CI: 0.37-1.47]) and the 18-month PFS rate was numerically higher in zanubrutinib (82.9% vs. 78.7%). In R/R MCL patients, naïve comparison showed investigator-assessed ORR was similar (83.7% vs. 87.9%; risk difference, -4.2% [95% CI: -14.8%-6.0%]), and CR rate was significantly higher in zanubrutinib over orelabrutinib (77.9% vs. 42.9%; risk difference, 35.0% [95% CI: 14.5%, 53.7%]). Investigator-assessed PFS was similar with a favorable trend (HR, 0.77 [95% CI: 0.45-1.32]) in zanubrutinib over orelabrutinib and the 12-month PFS rate was numerically higher in zanubrutinib (77.5% vs. 70.8%). MAIC result showed zanubrutinib demonstrated favorable PFS over orelabrutinib for R/R CLL/SLL patients. The naïve comparison showed zanubrutinib had favorable PFS and higher CR rate than orelabrutinib for R/R MCL patients.

**Supplementary Information:**

The online version contains supplementary material available at 10.1007/s10637-023-01376-1.

## Introduction

The B-cell receptor (BCR) signal pathway is a critical contributor to the survival and proliferation of malignant B cells [1, 2]. Inhibition of the BCR signal has been shown as a promising therapeutic method for B-cell malignancies [3]. Bruton’s tyrosine kinase (BTK), an essential member of the BCR signal pathway, plays an important role in the B-cell development involved in B-cell proliferation, maturation, differentiation, apoptosis, and migration [4]. Ibrutinib, a first-in-class BTK inhibitor, was first approved in the US in 2013 for the treatment of relapsed or refractory (R/R) mantle cell lymphoma (MCL) and has been a standard of care for naïve and R/R chronic lymphocytic leukemia/small lymphocytic lymphoma (CLL/SLL), R/R MCL, and R/R Waldenström macroglobulinemia (WM). However, the off-target kinase inhibition of ibrutinib, which was thought to cause diarrhea, bleeding, and atrial fibrillation [5], may limit its use as a treatment option. Highly selective, next-generation BTK inhibitors with fewer off-target effects are needed and being developed. Currently, several next-generation BTK inhibitors have been approved and launched, including acalabrutinib, zanubrutinib, and orelabrutinib. Zanubrutinib and orelabrutinib have been launched in China for the treatment of R/R CLL/SLL and R/R MCL.

Zanubrutinib, a novel next-generation BTK inhibitor, has greater selectivity for BTK and less off-target activity versus ibrutinib [6, 7]. Zanubrutinib was first approved in the US in 2019 as a breakthrough therapy for patients with MCL who received at least one prior therapy. It has received approval from China’s National Medical Products Administration (NMPA) for the treatment of R/R MCL, R/R CLL/SLL, and R/R WM. Two phase 3 head-to-head studies [8, 9] have demonstrated that zanubrutinib had less off-target toxicities (particularly cardiovascular toxicity) than ibrutinib in the treatment of WM and R/R CLL/SLL, and superior ORR and PFS over ibrutinib for R/R CLL/SLL [9, 10].

Orelabrutinib is also a novel next-generation BTK inhibitor with high selectivity for BTK [11] that has been only approved in China for the treatment of R/R CLL/SLL and R/R MCL.

BTK resynthesis was faster in patients with CLL than in healthy volunteers; therefore, it is hypothesized that complete and sustained BTK occupancy may improve efficacy outcomes [12]. Zanubrutinib has exposure coverage above its enzymatic half-maximal inhibitory concentration (IC50) during the entire dose interval for both twice daily and once daily dosing schedules. The ratio of C_trough_ (trough concentrations) /IC50 is 7 and 2 resulting in significantly higher concentrations than the IC50 during the entire 24-hr dosing period for both dosing schedules [13]. While the corresponding ratios of C_trough_/IC50 for orelabrutinib were estimated to be lower than 1 [14].

Because zanubrutinib and orelabrutinib are both approved and are widely used in China as treatment options for R/R CLL/SLL and R/R MCL, a further evaluation to understand the difference between these two novel BTKis is helpful for planning future research. In addition, with the absence of head-to-head studies, an indirect treatment comparison is an appropriate approach to evaluate the comparative efficacy of zanubrutinib and orelabrutinib based on different clinical trials. Currently, orelabrutinib only has available data on the Chinese patient population, thus the studies of zanubrutinib on the Chinese population were used in the indirect treatment comparison.

In the present study, we conducted two separate indirect comparisons to assess the efficacy of the two NMPA-approved next-generation BTK inhibitors zanubrutinib and orelabrutinib in the treatment of R/R CLL/SLL or R/R MCL patient based on Chinese populations.

## Methods

An unanchored matching-adjusted indirect comparison (MAIC) [15] was performed in patients with R/R CLL/SLL, and a naïve comparison was performed in patients with R/R MCL due to the different response assessment methodology and efficacy analysis set between the zanubrutinib and orelabrutinib trials. Individual patient data (IPD) from zanubrutinib studies (BGB-3111-205, BGB-3111-206) and aggregated data reported in publications from orelabrutinib studies (ICP-CL-00103, ICP-CL-00102) were used. Data were available from several cutoff dates of each included study. The data cutoff of the orelabrutinib study with the longest follow-up time of the primary endpoint was selected. The data cutoff of the zanubrutinib study with a comparable median follow-up time was selected for the comparisons.

## Data sources

### For R/R CLL/SLL

The data source of the efficacy of zanubrutinib in patients with R/R CLL/SLL was IPD, which is from the BGB-3111-205 study (NCT03206918). BGB-3111-205 [16, 17] is a single-arm, multicenter, phase II study of zanubrutinib in Chinese patients with R/R CLL/SLL. The primary endpoint was the overall response rate (ORR) assessed by an independent review committee (IRC). The longest data cutoff date of BGB-3111-205 used in this study was 1 December 2020, with a median follow-up time of 34 months [17], which was similar to the orelabrutinib study of ICP-CL-00103 study.

The literature review identified two publications [18, 19] of the ICP-CL-00103 study (NCT03493217) as the aggregated data source of orelabrutinib in patients with R/R CLL/SLL. The data cutoff date of the primary data source [18] of the ICP-CL-00103 study was 16 January 2021, which the IRC-assessed ORR was based on the longest follow-up (median follow-up: 25.6 months). The data cutoff date of the secondary data source of the ICP-CL-00103 study [19] was 10 August 2021, which the investigator-assessed ORR was based on the longest follow-up (median follow-up: 33.1 months). The ICP-CL-00103 study is an open-label, multicenter, single-arm phase II study of orelabrutinib in patients with R/R CLL/SLL. The primary endpoint was IRC-assessed ORR.

The eligibility criteria were similar between the BGB-3111-205 and ICP-CL-00103 studies, as shown in Supplementary Table 1.

### For R/R MCL

The data source of zanubrutinib was IPD from the BGB-3111-206 study (NCT03206970). BGB-3111-206 [20, 21] is a single-arm, open-label, phase II study of zanubrutinib in patients with R/R MCL. The primary endpoint was ORR assessed by an IRC. The data cutoff date of BGB-3111-206 used in this study was 15 February 2019 with a median follow-up time of 18.4 months [20], to achieve a similar median follow-up time as the ICP-CL-00102 study.

The literature review identified one publication [22] (data cutoff: 10 April 2020; median follow-up: 16.4 months) of the ICP-CL-00102 study (NCT03494179) as the aggregated data source of orelabrutinib. ICP-CL-00102 is a multicenter, open-label, phase II study of orelabrutinib in patients with R/R MCL. The primary endpoint was ORR assessed by an IRC.

The eligibility criteria were similar between the BGB-3111-206 and ICP-CL-00102 studies, as shown in Supplementary Table 2.

### Efficacy outcomes and assessment

Efficacy outcomes included IRC-assessed and investigator-assessed ORR and PFS.

### PFS

The definitions of PFS were similar across all four studies. PFS was defined as the time from the first dose of treatment to progression or death.

### ORR

#### For R/R CLL/SLL

The definitions of ORR were similar between the BGB-3111-205 and ICP-CL-00103 studies. For patients with R/R CLL/SLL, responses for patients with CLL included partial response (PR), PR with lymphocytosis, nodular PR, complete response (CR), or CR with incomplete hematologic recovery, and for patients with SLL, either PR or CR.

The response assessment methodology and efficacy analyses set were also similar between the two studies. The response assessment was according to the International Workshop on CLL guidelines [23] or the Lugano classification for SLL [24]. The efficacy analyses set included patients who received at least one dose of treatment.

#### For R/R MCL

The definitions of efficacy outcomes were similar between the BGB-3111-206 and ICP-CL-00102 studies. ORR was defined as either a PR or CR. However, the primary endpoint of IRC-assessed ORR was not reported in the ICP-CL-00102 study [22]; only investigator-assessed ORR was available. Moreover, the response assessment methodology and efficacy analyses set were different between the BGB-3111-206 and ICP-CL-00102 studies. Response evaluation was a PET-based assessment according to Lugano criteria [24] in the BGB-3111-206 study, while was a CT-based assessment according to Lugano criteria [24] in the ICP-CL-00102 study (CR was evaluated according to PET in only 28 out of 106 patients). The efficacy analysis set included all patients who received at least one dose of zanubrutinib, while the efficacy analysis set of the ICP-CL-00102 study excluded patients who were not evaluable due to protocol violation, adverse event (AE), or dropped out before the first assessment. The naïve comparison results will be conservative for zanubrutinib since the orelabrutinib trial excluded patients from the denominator in the process of estimating response rates; while those excluded unevaluable patients were included and counted as non-responders in the zanubrutinib trial.

## Study comparisons

### For R/R CLL/SLL

The study design, eligibility criteria, efficacy endpoints, and baseline characteristics showed sufficient similarities between the BGB-3111-205 and ICP-CL-00103 studies. The imbalances in the patient population between the two studies necessitated an MAIC to reduce bias when indirectly comparing zanubrutinib to orelabrutinib.

### For R/R MCL

The study design and eligibility criteria were similar between the BGB-3111-206 and ICP-CL-00103 studies. However, the response assessment methodology and efficacy analysis set were different between the BGB-3111-206 and ICP-CL-00102 studies. Therefore, a naïve comparison was conducted to indirectly and descriptively compare the efficacy of zanubrutinib versus orelabrutinib in patients with R/R MCL.

## Statistical analysis methods

### For R/R CLL/SLL

An unanchored MAIC was conducted in patients with R/R CLL/SLL. MAIC is a propensity-score-weighting-based method to generate comparative effectiveness evidence when IPD is available in one study and aggregate data in another [15]. In this analysis, the unanchored MAIC adjusts the mean of effect modifiers and prognostic factors in BGB-3111-205 to match those reported characteristics for ICP-CL-00103.

The first step when implementing a MAIC is to align the patient population of the trials to be compared. We excluded one SLL patient with Ann-Arbor stage Phase 1 and five CLL patients with Binet stage A from BGB-3111-205 to match the population of ICP-CL-00103. Patients across two trials were matched on available potential effect modifiers and prognostic variables, including age category, sex, ECOG performance status, bulky disease, IGHV unmutated, cytogenetic mutation (Del[17p] or *TP53* mutation, Del[11q], Trisomy 12), and the number of prior lines of treatment. The baseline characteristics to be matched in MAIC were selected based on the preliminary feasibility assessment and discussions with clinical experts. The weight of individual patients was calculated by the method of moments, in line with published guidance from the National Institute for Health and Care Excellence Decision Support Unit [25]. The weighted efficacy outcomes of BGB-3111-205 were compared with those reported in ICP-CL-00103. The survival outcomes of ICP-CL-00103 were estimated from the pseudo IPD generated from the digitized Kaplan-Meier curve and the at-risk table of ICP-CL-00103 [26]. We notice that the digitization process may introduce bias. Therefore, the reconstructed pseudo IPD was visually inspected by the plots of the estimated Kaplan-Meier curve versus read-in, the estimated numbers of patients at risk versus reported, and the estimated survival probabilities minus read-in survival probabilities over time by the R package *IPDfromKM* [26]. All validation plots showed the pseudo IPD was numerically accurate and thus the numerical error was almost negligible. The hazard ratio with a 95% confidence interval of the survival outcomes between two trials was estimated by a (weighted) Cox model. Risk differences with a 95% confidence interval of all binary outcomes between two trials were estimated by the Miettinen-Nurminen method.

### For R/R MCL

A naïve indirect comparison was performed in patients with R/R MCL due to the different response assessment methodology and efficacy analysis set between BGB-3111-206 and ICP-CL-00102. As only investigator-assessed outcomes were reported in the publication of the orelabrutinib trial, the investigator-assessed outcomes were used and compared for R/R MCL patients. The difference of important baseline characteristics and efficacy outcomes were estimated for descriptive purpose. The survival outcomes of ICP-CL-00102 were estimated by the same method as that for ICP-CL-00103. The hazard ratio and risk differences with a 95% confidence interval were estimated by the same methods as those for R/R CLL/SLL.

All analyses were performed using R version 4.0.2. The results reported in this paper are post hoc analysis result.

## Results

### Baseline characteristics

#### For R/R CLL/SLL

Before matching, the baseline characteristics were highly comparable between orelabrutinib and zanubrutinib (Table 1). Only the proportion of patients with IGHV unmutated was a little higher in the zanubrutinib group than in the orelabrutinib group (56% vs. 41%). After matching, the baseline characteristics were balanced between the two populations, with an effective sample size of 66 in the zanubrutinib group.

**Table 1:** Baseline characteristics of orelabrutinib versus zanubrutinib before and after matching in patients with R/R CLL/SLL

**Characteristics**	**ICP-CL-00103**	**BGB-3111-205**			
	Orelabrutinib (N=80)	Zanubrutinib			
		Before matching (N=85)^*^	*P* value	After matching (ESS=66)	*P* value
Age <65 years, %	72	66	0.45	72	1
Male, %	64	58	0.52	64	1
ECOG PS ≥1	94	96	0.65	94	1
Bulky disease LD ≥5 cm, %	42	42	1	42	1
Del(17p) or *TP53* mutation, %	22	26	0.75	23	1
Del(11q), %	24	21	0.83	24	1
IGHV unmutated, %	41	56	0.07	41	1
Trisomy 12, %	19	21	0.85	19	1
Prior lines of treatment <2, %	54	51	0.80	54	1

*CLL* chronic lymphocytic leukemia, *ECOG PS* Eastern Cooperative Oncology Group performance status, *ESS* effective sample size, *LD* longest diameter, *R/R* relapsed/refractory SLL small lymphocytic lymphoma

^*^One SLL patient with Ann-Arbor stage Phase 1 and five CLL patients with Binet stage A were excluded from BGB-3111-205 to match the population of ICP-CL-00103

### For R/R MCL

The baseline characteristics were comparable between orelabrutinib and zanubrutinib (Table 2). Patients in the zanubrutinib group had a higher proportion of ≥2 prior lines of treatment (71% vs. 55%) and a lower proportion of ECOG PS ≥1 (30% vs. 54%) compared with those in the orelabrutinib group. As the response assessment methodology and efficacy analysis set were different between the BGB-3111-206 and ICP-CL-00102 studies, a naïve comparison was conducted to assess the efficacy between zanubrutinib and orelabrutinib.

**Table 2:** Baseline characteristics of orelabrutinib versus zanubrutinib in patients with R/R MCL

**Characteristics**	**ICP-CL-00102**	**BGB-3111-206**	*P* value
	Orelabrutinib (N=106)	Zanubrutinib (N=86)	
Age <65 years, %	72	74	0.80
Male, %	79	78	0.96
ECOG PS ≥1	54	30	<0.01
Bulky disease LD ≥5 cm, %	39	45	0.43
Bone marrow involvement, %	41	45	0.60
Stage III-IV, %	94	91	0.49
Prior lines of treatment ≥2, %	55	71	0.03

*ECOG PS* Eastern Cooperative Oncology Group performance status, *LD* longest diameter, *MCL* mantle cell lymphoma, *R/R* relapsed/refractory

## Efficacy outcomes

### Response rates

#### For R/R CLL/SLL

Based on the IRC assessment, after matching, ORR was comparable between zanubrutinib and orelabrutinib (86.6% vs. 92.5%; risk difference, -5.9% [95% CI: -15.8%, 3.8%]; Table 3). CR rate was significantly different between zanubrutinib versus orelabrutinib (5.7% vs. 16.3%; risk difference, -10.5% [95% CI: -20.9%, -1.1%]).

**Table 3:** Efficacy outcomes assessed by IRC of zanubrutinib versus orelabrutinib in patients with R/R CLL/SLL

	**Zanubrutinib** **N=85/ESS=66**	**Orelabrutinib** **N=80**	**Risk difference/HR** **(95% CI)**
**Median follow-up time**, months	34	25.6	
**Response rates before matching**, % (95% CI)			
ORR	87.1 (78.0, 93.4)	92.5 (84.4, 97.2)	-5.4 (-15.3, 4.2)
CR^a^	5.9 (1.9, 13.2)	16.3 (8.9, 26.2)	-10.4 (-20.8, -0.9)
PR^b^	81.2 (71.2, 88.8)	66.4 (54.8, 76.4)	14.9 (1.5, 28.1)
**Response rates after matching**, % (95% CI)			
ORR	86.6 (77.5, 93.0)	92.5 (84.4, 97.2)	-5.9 (-15.8, 3.8)
CR^a^	5.7 (1.8, 13.0)	16.3 (8.9, 26.2)	-10.5 (-20.9, -1.1)
PR^b^	80.9 (70.9, 88.6)	66.4 (54.8, 76.4)	14.7 (1.2, 27.9)
**18-month PFS rate, % (95% CI)**			
Before matching	85.4 (75.6, 91.4)	78.7 (75.6, 86.6)	
After matching	82.9 (70.5, 90.4)	78.7 (75.6, 86.6)	
**PFS, median (95% CI) months**			
Before matching	NR	NR	0.69 (0.36-1.31)
After matching	NR	NR	0.74 (0.37-1.47)

*CLL* chronic lymphocytic leukemia, *CR* complete response *ESS* effective sample size, *HR* hazard ratio, *IRC* independent review committee, *NR* not reached, *ORR* overall response rate, *PR* partial response, *PFS* progression-free survival, *PR* partial response, *R/R* relapsed/refractory, *SLL* small lymphocytic lymphoma

^a^CR included CR and CR with incomplete bone marrow recovery (CRi). No patient was CRi in zanubrutinib, and 1.3% of patients were CRi in orelabrutinib

^b^PR included PR and PR with lymphocytosis

Based on the investigator’s assessment, after matching, ORR was also comparable between zanubrutinib versus orelabrutinib (90.1% vs. 93.8%; risk difference, -3.7% [95% CI: -12.8%, 5.2%]). CR rate was significantly lower in zanubrutinib compared with orelabrutinib (11.0% vs. 26.3%; risk difference, -15.2% [95% CI, -27.2%, -3.4%]).

### For R/R MCL

The naïve comparison showed that ORR assessed by the investigator was similar between zanubrutinib and orelabrutinib (83.7% vs. 87.9%; risk difference, -4.2 [95% CI: -14.8%, 6.0%]). CR rate was significantly higher in zanubrutinib compared with orelabrutinib (77.9% vs. 42.9%; risk difference, 35.0% [95% CI: 14.5%, 53.7%]; Table 4).

**Table 4:** Efficacy outcomes assessed by investigator of zanubrutinib versus orelabrutinib in patients with R/R MCL

**% (95% CI)**	**Zanubrutinib**^**a**^ **N=86**	**Orelabrutinib**^**b**^ **N=99**	**Risk difference/HR (95% CI)**
Median follow-up time, months	18.4	16.4	
ORR	83.7 (74.2, 90.8)	87.9 (79.8, 93.6)	-4.2 (-14.8, 6.0) ^d^
CR	77.9 (67.7, 86.1)	42.9 (24.5, 62.8) ^c^	35.0 (14.5, 53.7)
		34.3 (25.0, 43.8)	
PR	5.8 (1.9, 13.0)	53.5 (43.2, 63.6)	-47.7 (-58.2, -36.2) ^d^
6-month PFS rate, % (95% CI)	86.7 (77.2, 92.4)	84.9 (76.1, 90.6)	
12-month PFS rate, % (95% CI)	77.5 (66.6, 85.2)	70.8 (60.5, 78.9)	
PFS, median (95% CI) months	NR	NR (17.3, NR)	0.77 (0.45-1.32) ^d^

*CR* complete response, *HR*, hazard ratio, *MCL*, mantle cell lymphoma, *NR*, not reached, *ORR*, overall response rate, *PFS*, progression-free survival, *PR*, partial response, *R/R*, relapsed/refractory

^a^Response evaluations were based on PET-CT. Efficacy analyses included patients who received at least one dose of zanubrutinib (n=86)

^b^Response evaluations were based on CT. Efficacy analyses included 99 evaluable patients; seven patients were not evaluable due to protocol violation, adverse event or dropped out before first assessment

^c^CR was evaluated based on PET (n=28)

^d^The response assessment methodology is different between trials, the risk difference of ORR and PR are descriptive and for reference only, and the HR of PFS is descriptive and for reference only

## Survival outcomes

### For R/R CLL/SLL

**Fig. 1:**
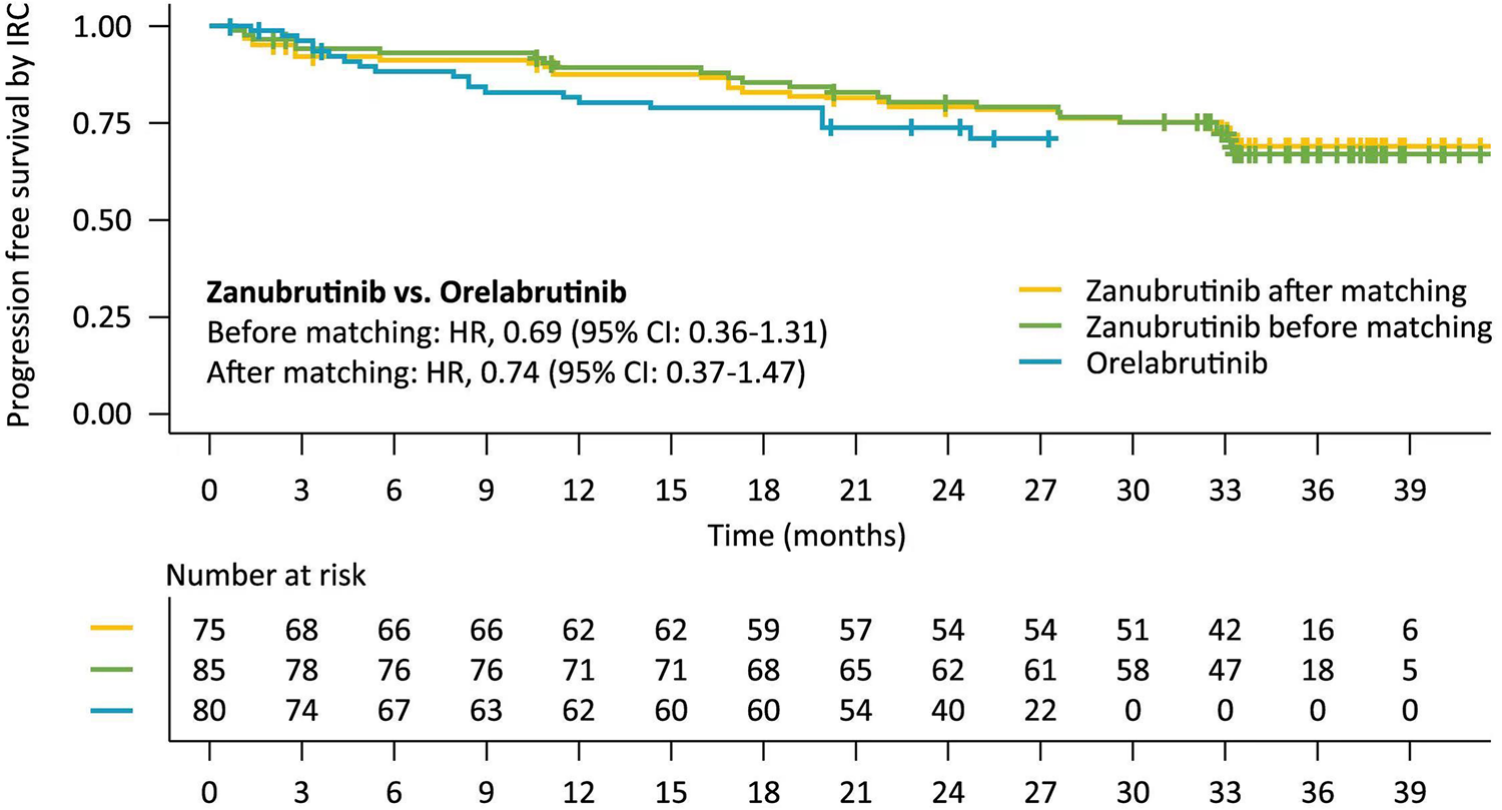
Progression-free survival in R/R CLL/SLL patients assessed by IRC before and after matching

As the survival outcome assessed by IRC, with the median follow-up time of 34 months in zanubrutinib and 25.6 months in orelabrutinib, PFS was not reached (NR) in both groups but was more favorable in zanubrutinib compared with orelabrutinib with a hazard ratio (HR) of 0.74 (95% CI, 0.37-1.47) after matching (Table 3; Fig. 1). The unadjusted comparison had the consistent result that PFS was favorable in zanubrutinib compared with in orelabrutinib (median PFS: NR vs. NR; HR, 0.69 [95% CI: 0.36-1.31]). The 18-month PFS rate was numerically higher in zanubrutinib than in orelabrutinib (82.9% vs. 78.7%) after matching.

The survival outcomes assessed by the investigator were also analyzed. With the median follow-up time of 34 months in zanubrutinib and 33.1 months in orelabrutinib, zanubrutinib had favorable PFS compared with orelabrutinib (median PFS: NR vs. NR; HR, 0.84 [95% CI: 0.47-1.53]) after matching. The 30-month PFS rate was also numerically higher in zanubrutinib than in orelabrutinib (73.6% vs. 69.7%) after matching.

### For R/R MCL

**Fig. 2:**
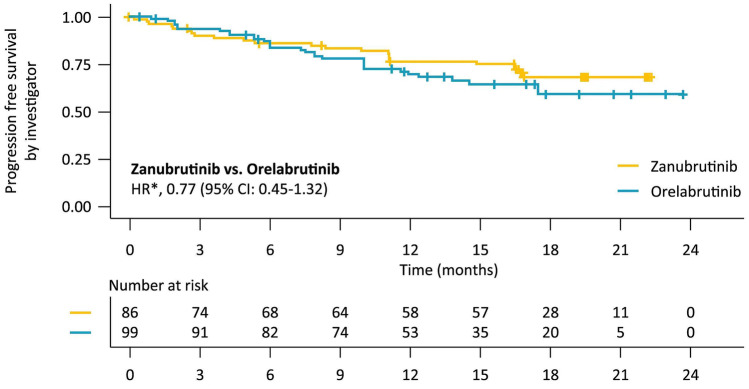
Progression-free survival in R/R MCL patients assessed by the investigator ^*^ The HR of PFS is descriptive and for reference only

With the median follow-up time of 18.4 months in zanubrutinib and 16.4 months in orelabrutinib, the PFS assessed by the investigator was favorable in zanubrutinib compared with orelabrutinib (median PFS: NR vs. NR; HR, 0.77 [95% CI: 0.45-1.32]; Table 4; Fig. 2). The 12-month PFS rate was also numerically higher in zanubrutinib than in orelabrutinib (77.5% vs. 70.8%).

## Discussion

CLL/SLL are slow-growing types of indolent non-Hodgkin Lymphoma. The ultimate treatment goal of CLL/SLL is to achieve a longer overall survival (OS), while minimizing toxicities and improving the quality of life. In the absence of a survival benefit, achieving a long PFS is a reasonable goal of therapy [27]. For patients with CLL/SLL, continuous treatment with a single-agent BTK inhibitor significantly improved PFS and OS versus standard chemotherapy and/or chemoimmunotherapy [28–31]. Moreover, the interim analysis of a head-to-head study (ALPINE) showed an improved PFS in patients with R/R CLL/SLL treated with zanubrutinib compared with ibrutinib [9], and the final analysis showed that zanubrutinib is superiority to ibrutinib in PFS (HR: 0.65 [95% CI, 0.49-0.86]; *P* = 0.0024) in patients with R/R CLL/CLL [10]. There is no head-to-head study comparing any two next-generation BTK inhibitors. Shadman et al found that zanubrutinib showed higher selectivity than ibrutinib and acalabrutinib, and comparable selectivity to orelabrutinib. In addition, zanubrutinib had a lower IC_50_ value (0.71 vs. 15 nM) against BTK compared with orelabrutinib [14], which might contribute to the favorable efficacy of zanubrutinib over orelabrutinib. In this study, with the median follow-up of 34 months in zanubrutinib and 25.6 months in orelabrutinib, ORR was comparable, and median PFS was not reached but demonstrated a favorable trend on PFS for zanubrutinib over orelabrutinib in R/R CLL/SLL patients (IRC and investigator-assessed PFS HR of 0.74 and 0.69, respectively). No significant difference in PFS was observed, which might be due to the limited sample size and the relatively short follow-up time. Further updated analyses with a long follow-up time may help to consolidate these results. What we have found in this study was supported by a network meta-analysis [32], which aimed to estimate the efficacy of zanubrutinib versus standard of treatment for R/R CLL. Results suggested a remarkable improvement in PFS for zanubrutinib over acalabrutinib (HR, 0.52 [95% CI: 0.30-0.90]) and a trend favoring zanubrutinib over acalabrutinib in OS (HR, 0.75 [95% CI: 0.35-1.59]). These findings indicate that PFS on zanubrutinib might be superior to other next-generation BTK inhibitors for patients with R/R CLL/SLL.

R/R MCL has historically poor long-term survival compared with other B-cell malignancies [33]. In addition to survival outcomes, the treatment strategies for R/R MCL tend to focus on the relief of patients’ symptoms and their response. Ibrutinib was approved by the FDA for R/R MCL based on results of a phase 2 trial with a long-term ORR of 78% and CR of 27% [34]. The FDA approved zanubrutinib for R/R MCL when it showed an ORR of 83.7% and CR of 77.9% [35]. There is no study on the comparison of different next-generation BTK inhibitors. In this study, we conducted a naïve indirect treatment comparison due to the different response evaluation methodology between zanubrutinib and orelabrutinib studies (a PET-based assessment in the zanubrutinib study while a CT-based assessment in the orelabrutinib study). With the short median follow-up time of 18.4 months in zanubrutinib and 16.4 months in orelabrutinib, zanubrutinib has shown a favorable PFS trend over orelabrutinib with the HR of 0.77. CR rate was also significantly higher in zanubrutinib than in orelabrutinib.

We did not analyze the AEs in this study considering the potential differences in AE collection, reporting, follow-up time between studies, and limited sample size.

With the growing number of approved next-generation BTK inhibitors, it is important to understand the potential differences between them. The findings in the present study may provide useful information for future study.

However, there are several limitations in this study. In the R/R CLL/SLL analysis, because both included trials are single-arm trials, the comparison was conducted through an unanchored MAIC analysis, which relies on the assumption that all prognostic factors and effect modifiers are identified and included in population matching. Despite all available prognostic factors and effect modifiers having been included to reduce the bias, this assumption is still strong and can never be verified. Potential violation of this assumption leads to bias. In the R/R MCL analysis, the response assessment methodology and efficacy analysis set were different between the two included trials. Therefore, only a naïve comparison was conducted, and all the results are descriptive. However, the results of both analyses, even though they are likely biased, generated signals and hypotheses for further research.

## Conclusions

In conclusion, MAIC results showed that zanubrutinib demonstrated a comparable ORR but more favorable PFS compared with orelabrutinib for R/R CLL/SLL patients, and the naïve comparison showed that zanubrutinib had a higher CR rate and favorable PFS over orelabrutinib for R/R MCL patients.

## Supplementary Information

Below is the link to the electronic supplementary material.

Supplementary file1 (DOCX 22 kb)

Supplementary file2 (DOCX 22 kb)

## Data Availability

The datasets used are available from the corresponding author on reasonable request.
