# Supplementary material for: Indirect comparisons of efficacy of zanubrutinib versus orelabrutinib in patients with relapsed or refractory chronic lymphocytic leukemia/small lymphocytic lymphoma or relapsed or refractory mantle cell lymphoma
Source: Invest New Drugs. 2023 Jul 8;41(4):606–16. doi: 10.1007/s10637-023-01376-1 (PMC10447591; doi:10.1007/s10637-023-01376-1)
Supplement: Supplementary file 1 — Supplementary file1 (DOCX 22 kb) [file 10637_2023_1376_MOESM1_ESM.docx]

**Supplementary Table 1.** Study design characteristics and inclusion criteria of BGB-3111-205 versus ICP-CL-00103

|  | BGB-3111-205 | ICP-CL-00103 |
| --- | --- | --- |
| Study phase | II | II |
| Blinding | Open label | Open label |
| Primary endpoint | ORR by IRC | ORR by IRC  ORR by IRC not reported in the long follow-up publication, ORR by investigator with longer follow-up will be also analyzed in this study. |
| Median duration of follow-up | 34 months | 25.6 months by IRC; 33.1 months by investigator |
| Database cut-off date | 2020-12-01 | 2021-01-16 (IRC-assessed ORR); 2021-08-10 (INV-assessed ORR) |
| Key eligibility criteria |  |  |
|  | Age ≥18 years | Age ≥18 years old |
|  | CLL or SLL, as defined by iwCLL2008, met requirement for treatment | CLL/SLL with at least one treatment  indication by iwCLL2008 |
|  | ECOG PS ≤2 | ECOG PS ≤2 |
|  | Must have had relapsed/refractory disease after a minimum of one prior line of a standard chemotherapy regimen (e.g., fludarabine or chlorambucil-based) administered over at least two cycles | Must have received ≥1 prior  treatment |
