# Supplementary material for: Indirect comparisons of efficacy of zanubrutinib versus orelabrutinib in patients with relapsed or refractory chronic lymphocytic leukemia/small lymphocytic lymphoma or relapsed or refractory mantle cell lymphoma
Source: Invest New Drugs. 2023 Jul 8;41(4):606–16. doi: 10.1007/s10637-023-01376-1 (PMC10447591; doi:10.1007/s10637-023-01376-1)
Supplement: Supplementary file 2 — Supplementary file2 (DOCX 22 kb) [file 10637_2023_1376_MOESM2_ESM.docx]

**Supplementary Table 2.** Study design characteristics and inclusion criteria of BGB-3111-206 versus ICP-CL-00102

|  | BGB-3111-206 | ICP-CL-00102 |
| --- | --- | --- |
| Study phase | II | II |
| Blinding | Open label | Open label |
| Primary endpoint | ORR by IRC | ORR by IRC; ORR by IRC was not reported in publication, ORR by INV was used as primary endpoint in this study |
| Median duration of follow-up | 18.4 months | 16.4 months by investigator |
| Database cut-off date | 2019-02-15 | 2020-04-10 |
| Key eligibility criteria |  |  |
|  | MCL with cyclin D1 overexpression or t(11; 14) | MCL with either t(11;14) and/or cyclin D1 overexpression |
|  | Patients had received at least one prior line of therapy and had relapsed or were refractory (i.e., achieved neither partial nor complete response) to their last  regimen. | Refractory or relapsed mantle cell lymphoma who has received at least 1 but no more than 4 prior therapies for MCL |
|  | ECOG PS 0-2 | ECOG PS 0-2 |
|  | Achieved neither partial nor complete response to their last regimen | Documented failure to achieve at least partial response or documented disease progression after response to the most recent treatment regimen |
